# Akebia saponin D acts via the PPAR‐gamma pathway to reprogramme a pro‐neurogenic microglia that can restore hippocampal neurogenesis in mice exposed to chronic mild stress

**DOI:** 10.1111/cns.14196

**Published:** 2023-03-29

**Authors:** Jinqiang Zhang, Qin Liu, Dapeng Su, Liangyuan Li, Chenghong Xiao, Hui He, Zili You, Tao Zhou

**Affiliations:** ^1^ Resource Institute for Chinese & Ethnic Materia Medica Guizhou University of Traditional Chinese Medicine Guiyang China; ^2^ School of Life Science and Technology University of Electronic Science and Technology of China Chengdu China

**Keywords:** akebia saponin D, BDNF–TrkB, major depressive disorder, microglia, neurogenesis, peroxisome proliferator‐activated receptor‐γ

## Abstract

**Background:**

Using drugs to modulate microglial function may be an effective way to treat disorders, such as depression, that involve impaired neurogenesis. Akebia saponin D (ASD) can cross the blood–brain barrier and exert anti‐inflammatory and neuroprotective effects, so we wondered whether it might influence adult hippocampal neurogenesis to treat depression.

**Methods:**

We exposed C57BL/6 mice to chronic mild stress (CMS) as a model of depression and then gave them ASD intraperitoneally once daily for 3 weeks. We investigated the effects of ASD on microglial phenotype, hippocampal neurogenesis, and animal behavior. The potential role of the peroxisome proliferator‐activated receptor‐gamma (PPAR‐γ) or BDNF–TrkB pathway in the pro‐neurogenesis and anti‐depressant of ASD was identified using there inhibitors GW9662 and K252a, respectively. The neurogenic effects of ASD‐treated microglia were evaluated using conditioned culture methods.

**Results:**

We found that CMS upregulated pro‐inflammatory factors and inhibited hippocampal neurogenesis in dentate gyrus of mice, while inducing depressive‐like behaviors. Dramatically, ASD (40 mg/kg) treatment reprogrammed an arginase (Arg)‐1^+^ microglial phenotype in dentate gyrus, which increased brain‐derived neurotrophic factor (BDNF) expression and restored the hippocampal neurogenesis, and partially ameliorated the depressive‐like behaviors of the CMS‐exposed mice. K252a or neurogenesis inhibitor blocked the pro‐neurogenic, anti‐depressant effects of ASD. Furthermore, ASD activated PPAR‐γ in dentate gyrus of CMS mice as well as in primary microglial cultures treated with lipopolysaccharide. Blocking the PPAR‐γ using GW9962 suppressed the ASD‐reprogrammed Arg‐1^+^ microglia and BDNF expression in dentate gyrus of CMS mice. Such blockade abolished the promoted effects of ASD‐treated microglia on NSPC proliferation, survival, and neurogenesis. The pro‐neurogenic and anti‐depressant effects of ASD were blocked by GW9962.

**Conclusion:**

These results suggested that ASD acts via the PPAR‐γ pathway to induce a pro‐neurogenic microglia in dentate gyrus of CMS mice that can increase BDNF expression and promote NSPC proliferation, survival, and neurogenesis.

## Graphical abstract

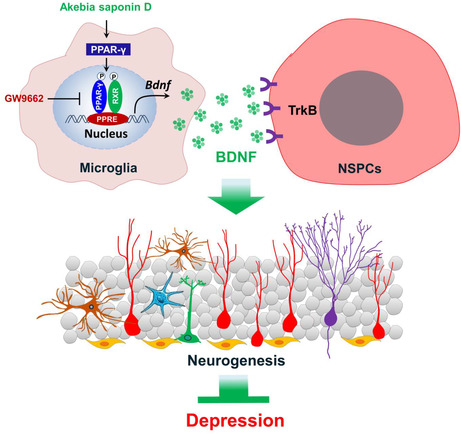

## 1 INTRODUCTION

Major depressive disorder affects millions of people worldwide and has multifactorial causes, leading to heterogeneous clinical presentations.^1^, ^2^ Current anti‐depressant medications often lack efficacy,^3^, ^4^ highlighting the need for further studies of how depression and related diseases can be treated.

The occurrence and development of depression have been linked to decreased neurogenesis in the adult hippocampus.^5^, ^6^, ^7^ Restoring hippocampal neurogenesis can enhance stress resistance and mitigate depressive symptoms,^8^, ^9^ so this may be an effective strategy for treating depression.^10^, ^11^ During neurogenesis in adult hippocampus, neural stem/progenitor cells (NSPCs) proliferate and differentiate into neurons.^12^ In neurogenic niches, NSPCs produce neurons that support learning, memory, and behavior, and this production continues through adulthood.^13^ Efficient neurogenesis depends on a supportive “neurogenic niche” in the hippocampus.^9^

An important modulator of neurogenesis in this niche is microglia.^14^, ^15^, ^16^ Pro‐inflammatory microglia suppress adult hippocampal neurogenesis, while anti‐inflammatory microglia do the opposite.^9^, ^17^, ^18^ Chronic stress hyperactivates microglial cells, which secrete inflammatory mediators that impair neuroplasticity and neurogenesis.^19^, ^20^, ^21^ Nevertheless, microglia are endowed with phenotypic plasticity to regulate physiological responses and behavioral outcomes during stress.^22^, ^23^, ^24^, ^25^ In particular, the Arg‐1^+^ microglia were considered a neuroprotective microglial phenotype.^26^, ^27^ Our previous research found that this subgroup of microglia can secrete brain‐derived neurotrophic factor (BDNF) to promote hippocampal neurogenesis in responding to chronic stress, helping protect against depressive‐like symptoms.^9^ Thus, pharmacological modulation of the microglial phenotype may allow control of NSPC proliferation and differentiation to treat disorders associated with neurogenic dysfunction.

Several natural products show promise for modulating microglial phenotype.^21^ For example, the akebia saponin D (ASD), a triterpenoid saponin, is abundant in the traditional Chinese medicine Radix Dipsaci, which exerts anti‐osteoporotic, anti‐inflammatory, and neuroprotective effects.^28^, ^29^, ^30^ Studies have shown that ASD can efficiently cross the blood–brain barrier^28^ and exerts neuroprotective effects.^31^ We have shown that ASD regulates microglial function to ameliorate neuroinflammation and depression‐like behaviors of mice exposed to lipopolysaccharide (LPS).^32^, ^33^ However, we are unaware of studies exploring whether ASD influences hippocampal neurogenesis, which may thereby help explain its antidepressant effects.

Thus, here we investigated the effects of ASD on microglial phenotypes, hippocampal neurogenesis, and depressive‐like behaviors in mice exposed to chronic mild stress (CMS). To gain greater mechanistic insights, we also examined the effects of conditioned medium from ASD‐induced microglia on NSPC proliferation, survival, and neuronal differentiation.

## 2 MATERIALS AND METHODS

### 2.1 Animals

Male C57BL/6J mice (7–8 weeks old) were purchased from Changsha Tianqin Biotechnology, caged individually, and assigned unique numbers. The mice were habituated to their new environment for 1 week. The mice were then habituated to a 1% sucrose solution for 48 h. Sucrose preference and body weight were determined weekly as described in Section 2.4.1. Body weight and sucrose preference during the first 3 weeks served as the pretreatment baseline, and animals were allocated into seven groups as described in Section 2.3.1. All experiments were approved by the Institutional Animal Care and Use Committee at the Guizhou University of Traditional Chinese Medicine.

### 2.2 Chronic mild stress (CMS)

Animals were exposed to CMS for 6 weeks as described.^9^ Everyday animals were exposed to two to three of the following stressors in random order: empty water bottles (12 h), food deprivation (12 h), tail clipping (10 min), restraint (2 h), lights‐off for 3 h during the daylight phase, cage shaking (1 h), cage tilting (45°, 24 h), reversal of the light–dark cycle (24 h), strobe lighting (12 h), damp bedding (24 h), and a soiled cage (24 h).

### 2.3 Pharmacological treatments in vivo

#### 2.3.1 Treatment with ASD and imipramine

Akebia saponin D (99.92% pure) was purchased from Chengdu Alfa Biotechnology and dissolved to a concentration of 4 mg/mL in 0.9% saline. After 4 weeks of CMS, the mice were allocated into seven groups such that the groups did not differ significantly in sucrose preference or body weight: control + saline (Ctrl), control + 40 mg/kg ASD (ASD), CMS + saline (CMS), CMS + 20 mg/kg ASD (CMS + ASD 20), CMS + 40 mg/kg ASD (CMS + ASD 40), CMS + 80 mg/kg ASD (CMS + ASD 80), and CMS + imipramine (CMS + IMI). The mice were intraperitoneally administered saline, ASD (20, 40 or 80 mg/kg/day), or imipramine (10 mg/kg/day; Sigma‐Aldrich) once daily (at 16:00 h) for 3 weeks. Based on the antidepressant effects of imipramine involved in hippocampal neurogenesis,^34^ imipramine was used as a positive control in this study. The doses of ASD and imipramine were chosen based on previous studies.^21^, ^32^

#### 2.3.2 Treatment with temozolomide

The potential role of the neurogenesis in the anti‐depressant was analyzed using the neurogenesis inhibitor temozolomide (TMZ, Sigma‐Aldrich). TMZ was dissolved in 0.9% saline containing 5% dimethyl sulfoxide (DMSO) at a concentration of 2.5 μg/mL. After 3 weeks of CMS, four animal groups with similar sucrose preference and body weight were formed: control + saline + DMSO (Ctrl), CMS + saline + DMSO (CMS), CMS + ASD + DMSO (CMS + ASD), and CMS + ASD + TMZ (CMS + ASD + TMZ). The mice were intraperitoneally administered saline, DMSO, ASD (40 mg/kg/day), and TMZ (25 μg/kg/day) once daily (at 16:00 h) for 3 weeks.

#### 2.3.3 Treatment with K252a or GW9662

To investigate the role of peroxisome proliferator‐activated receptor‐gamma (PPAR‐γ) pathway in ASD regulation of microglia phenotype, we used the PPAR‐γ inhibitor GW9662 (Sigma‐Aldrich). To investigate the potential role of the BDNF–tropomyosin receptor kinase B (TrkB) pathway in pro‐neurogenic effects of ASD, the K252a (Sigma‐Aldrich) was used to block the TrkB. GW9662 or K252a was dissolved in 0.9% saline containing 5% dimethyl sulfoxide (DMSO) at a concentration of 1 mg/mL and 2.5 μg/mL, respectively. After 3 weeks of CMS, five animal groups with similar sucrose preference and body weight were formed: control + saline + DMSO (Ctrl), CMS + saline + DMSO (CMS), CMS + ASD + DMSO (CMS + ASD), CMS + ASD + K252a (CMS + ASD + K252a), and CMS + ASD + GW9662 (CMS + ASD + GW9662). The mice were intraperitoneally administered saline, DMSO, ASD (40 mg/kg/day), and K252a (25 μg/kg/day) or GW9662 (1 mg/kg/day) once daily (at 16:00 h) for 3 weeks. The doses of K252a and GW9662 were chosen based on previous studies.^9^, ^35^

### 2.4 Behavioral testing

#### 2.4.1 Sucrose preference test (SPT)

The sucrose preference test was performed as described.^36^ Mice were individually housed, deprived of food and water for 12 h, and then given access to 1% sucrose solution (A) and water (B) for 2 h. The bottle positions were switched daily to avoid a side bias. The sucrose preference was calculated each week for each mouse using the formula: 100 × [VolA/(VolA + VolB)]. The sucrose consumption was normalized to body weight for each mouse.

#### 2.4.2 Forced swimming test (FST)

At 24 h before the test, each mouse was placed individually for 10 min in a glass cylinder (height, 25 cm; diameter, 15 cm) that was filled with water to a depth of 15 cm at 26°C. The next day, the mice were placed again in the same situation for 6 min. An observer masked to treatment conditions recorded the latency between suspension and first abandonment of struggle as well as the time spent immobile during the last 4‐min period.

#### 2.4.3 Open field test (OFT)

Mice were placed into an open field (50 × 50 cm^2^) and allowed to explore freely for 15 min. Total distance and time spent in the center (25 × 25 cm^2^) were quantified using video tracking software (OFT100, Taimeng Tech).

### 2.5 Primary culture of microglia and treatments

Primary microglia were isolated from brains of neonatal C57BL/6 mice (P0–P3) as described.^21^ The purified microglial cells were cultured at 37°C in DMED–F12 medium (Gibco) containing 10% fetal bovine serum (Gibco). After 7 days, microglia were pre‐treated with 10, 50, or 100 μM ASD (Alfabiotech) or pioglitazone (10 μM, Sigma‐Aldrich).^37^ After 30 min, microglia were treated for 24 or 48 h with either 50 ng/mL LPS (Sigma‐Aldrich) or phosphate‐buffered saline (PBS; BOSTER). Experimental groups were as follows: control group (Ctrl), not treated with ASD or LPS; LPS group (LPS), treated with LPS but not ASD; LPS + ASD (10, 50, 100 μM) group, treated with LPS and ASD at the indicated concentrations; and LPS + pioglitazone group, treated with LPS and 10 μM pioglitazone. At each time point, microglia were collected and transferred to new plates for further experiments.

### 2.6 Culture of NSPCs with conditioned medium from pre‐treated microglia

NSPCs were obtained from the hippocampus of 8‐week‐old male C57BL/6J mice as described.^38^ Microglia were plated at a density of 5 × 10^5^ cells/cm^2^, treated with either LPS or PBS for 24 h in the presence or absence of ASD, washed twice with PBS, and then cultured for another 24 h in DMEM‐F12 + GlutaMax medium (Gibco). The microglial medium was collected and used as conditioned medium (CM) to stimulate NSPC differentiation and proliferation.

NSPCs were cultured in CM from microglia treated with PBS, LPS, or both ASD and LPS. Experimental groups were as follows: PBS‐M‐CM group, incubated in CM from PBS‐treated microglia; LPS‐M‐CM group, incubated in CM from LPS‐treated microglia; ASD‐M‐CM group, incubated in CM from microglia treated with LPS and 50 μM ASD; and the Piog‐M‐CM group, incubated in CM from microglia treated with LPS and pioglitazone. NSPC proliferation, survival, and neuronal differentiation were evaluated using immunofluorescence as described in Section 2.13.

### 2.7 Treatment with K252a or GW9662 in vitro

In some experiments involving blockade of PPAR‐γ signaling pathway in ASD‐treated primary microglia, microglia were treated with ASD (50 μM) and PPAR‐γ inhibitor GW9662 (10 μM).^35^ After 30 min, microglia were treated for 24 h with either 100 ng/mL lipopolysaccharide (LPS; Sigma‐Aldrich) or phosphate‐buffered saline (PBS; BOSTER). Following the immunocytochemistry, RT‐PCR analysis, Western blot analysis, and conditional culture of NSPC were performed.

In some experiments involving blockade of BDNF–TrkB signaling pathway in NSPCS, BDNF receptor antagonist K252a (100 ng/mL)^9^ were added to the conditioned medium from the microglia treated by ASD and LPS. Then, the conditioned medium was used for proliferation culture or differentiation culture of NSPC.

### 2.8 BrdU incorporation

To determine NSPC proliferation and differentiation in the brain, mice received intraperitoneal injections of 5′‐bromo‐2′deoxyuridine (BrdU; Sigma‐Aldrich; 50 mg/kg/day) for 7 days.^9^ Mice were euthanized at 7 days after the last injection. To examination neuronal survival in the granular layer, animals were injected with a double dose of BrdU and euthanized at 7 weeks after injection.

To determine the NSPCs and newborn neurons survival during culture in conditioned medium from microglia activated with LPS in the presence or absence of ASD, the NSPCs were incubated with BrdU (100 ng/mL) for 24 h in proliferation medium.^9^ After that, these NSPCs were allowed to grow for 7 days in differentiation medium. Half of the volume of culture medium for induced differentiation was replaced with the microglia‐conditioned medium (M‐CM). After 3 days, the survival of the newborn neurons was measured using immunofluorescence as described in Section 2.13.

### 2.9 Animal perfusion

Mice were anesthetized with 10% pentobarbital and transcardially perfused with phosphate‐buffered saline (PBS) containing heparin. Brains were removed, fixed in 4% paraformaldehyde for 48 h, and washed with PBS, and we used the 30% sucrose to provide cryoprotection against the damaging water crystals formed around 0°C.^21^ Coronal sections containing the hippocampus were obtained using a cryostat slicer (CM1900; Leica Microsystems). Six sequential slices were collected into each well of a 12‐well plate containing PBS with 0.02% sodium azide and stored at 4°C. The 20‐μm‐thick slices were used for immunofluorescence, and the 100‐μm‐thick slices were used for protein and RNA extraction.

### 2.10 RNA extraction and real‐time PCR (RT‐PCR)

The dentate gyrus was isolated form slices containing the hippocampus. Total RNA was isolated from dentate gyrus or cultured cells using Trizol (Invitrogen Life Technologies) according to the manufacturer's instructions. RT‐PCR was performed using the First Strand cDNA Synthesis Kit (TaKaRa) according to the manufacturer's instructions. RT‐PCR amplification was performed using a Bio‐Rad CFX 96 system (Bio‐Rad Laboratories) and the primers in Table S1. Each sample was tested in triplicate. The threshold cycle (Ct) number was determined from the linear phase of the amplification plot using the −ΔΔ*C*_t_ method, and values were normalized against the housekeeping gene β‐actin.

### 2.11 Enzyme‐linked immunosorbent assay (ELISA)

The dentate gyrus was dissociated from slices containing the hippocampus, flash‐frozen in liquid nitrogen, and homogenized. Primary microglia were cultured in six‐well plates at 5 × 10^5^ cells/cm^2^ and then treated for 24 or 48 h with LPS or PBS in the presence or absence of ASD. The culture medium was collected, microglia were lysed in cell lysis buffer (Solarbio), and the lysates were centrifuged at 1000 *g* for 30 min. The concentration of total protein in the supernatant was determined using the BCA kit (BOSTER), and each sample was diluted to 1 g/mL. Then, samples were assayed using commercial ELISAs against the following signaling factors: interleukin (IL)‐1β, tumor necrosis factor (TNF)‐α, IL‐10, IL‐4, insulin‐like growth factor (IGF)‐1, and brain‐derived neurotrophic factor (BDNF; BOSTER); inducible nitric oxide synthase (iNOS) and arginase (Arg‐1; Elabscience); transforming growth factor (TGF)‐β (4A Biotech); and basic fibroblast growth factor (bFGF), epidermal growth factor (EGF; ColorfulGene Biotech), and nerve growth factor (NGF; BOSTER). The manufacturer‐specified detection limit of all kits was 1–4 pg/mL.

### 2.12 Western blotting (WB)

Mice were anesthetized with 10% pentobarbital and transcardially perfused with 0.9% NaCl. Hippocampi were removed and homogenized. Cultures of proliferative NSPCs were sonicated in RIPA buffer containing protease and phosphorylase inhibitors (Solarbio), lysates were centrifuged at 1000 *g* for 30 min, and equal amounts of soluble protein were fractionated by sodium dodecyl sulphate–polyacrylamide gel electrophoresis and transferred to PVDF membranes. Membranes were washed with Tris‐buffered saline containing 0.1% Tween‐20 (TBST), incubated with skim milk for 30 min, then incubated on a shaker at 4°C overnight with primary antibodies as listed in Table S2. Membranes were washed three times with TBST, incubated for 30 min with secondary antibody (1:10,000; Abcam), washed three times with TBST, and then developed for 1–2 min using enhanced chemiluminescence (Millipore, MMAS). Blots were visualized using the ChemiDoc Touch system (Bio‐Rad), and band intensity was quantified using Alpha software (version 1.45 J; National Institutes of Health).

### 2.13 Immunohistochemistry and immunocytochemistry

The following types of cells were plated separately at a density of 10^5^ cells/cm^2^ and cultured for 24 h: microglia, neurospheres, proliferative NSPCs, and differentiated NSPCs. Cells were fixed with 4% paraformaldehyde (pH 7.2) for 30 min. Slices containing the hippocampus and culture cells were permeabilized with 0.5% Triton X‐100 in PBS for 15 min, blocked in 10% donkey serum (Solarbio) for 2 h, and then incubated overnight at 4°C with the following primary antibodies as listed in Table S3. Slices or cells were washed three times with PBS and then incubated for 2 h at room temperature with DyLight 549‐ or DyLight 488‐conjugate secondary antibodies (both 1:300; Jackson ImmunoResearch). Finally, cells were incubated for 5 min with 4′, 6‐diamidino‐2‐phenylindole (DAPI; 1:10,000, Roche) and imaged using a fluorescence microscope (Olympus IX 73).

### 2.14 Molecular docking

The potential binding of ASD to peroxisome proliferator‐activated receptor (PPAR)‐γ was explored in molecular docking studies using the Surflex‐Dock module in Sybyl‐X2.1 software (Tripos Associates), based on previous work.^39^

### 2.15 Imaging and statistical analyses

Images were analyzed as described.^9^ Statistical analyses were performed using GraphPad Prism (version 8.0, SPSS Inc.). Experimental data were expressed as mean ± SEM. Kolmogorov–Smirnov test was used to assess data distribution. For normally distributed data, one‐way ANOVA was used to analyze differences between multiple groups. Data that did not exhibit a normal distribution were analyzed using Kruskal–Wallis tests between multiple groups. Differences were considered statistically significant if *p* < 0.05.

## 3 RESULTS

### 3.1 Akebia saponin D ameliorates depressive‐like behaviors of CMS mice

**FIGURE 1:**
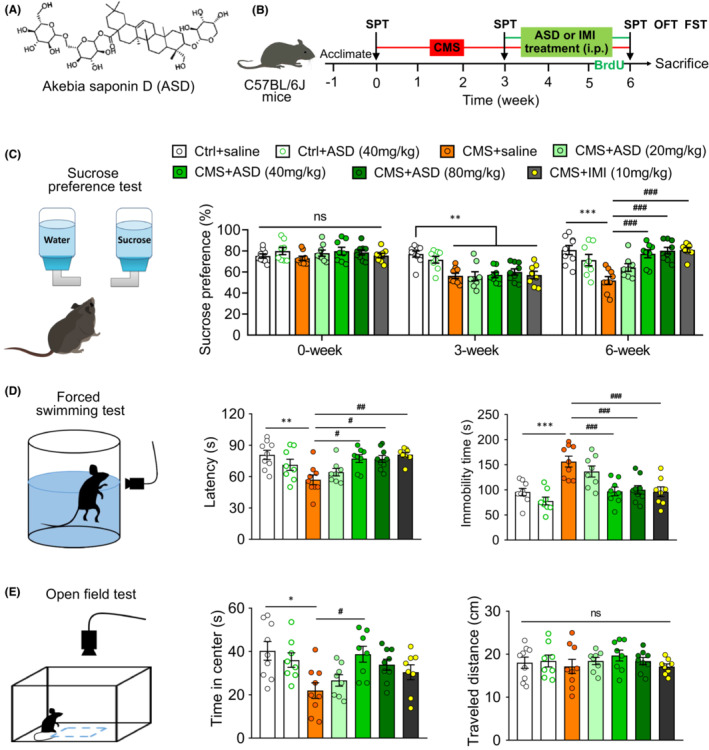
Akebia saponin D ameliorates CMS‐induced depressive‐like behaviors in mice. (A) The structure of akebia saponin D (ASD). (B) Scheme of the experimental procedure. ASD, chemical structural formula; CMS, chronic mild stress; IMI, imipramine; FST, forced swimming test; OFT, open field test. (C) Effects of ASD on the sucrose preference of mice. On the left is the diagrammatic figure for sucrose preference test. The histogram shows the change in sucrose preference on baseline (0 week), before treatment (3 weeks), or after treatment (6 weeks) with ASD or IMI in control (Ctrl) mice and mice subjected to CMS. (D) Effects of ASD on immobility time and latency of CMS mice in the forced swimming test. On the left is the diagrammatic figure for forced swimming test. The histogram shows the change in immobility time and latency in Ctrl mice and mice subjected to CMS, followed by treatment with ASD or IMI. (E) Effects of ASD on the time in the center and distance traveled by CMS mice in the open field test. On the left is the diagrammatic figure for open field test. The histogram shows the change in time in the center and distance traveled by Ctrl mice and mice subjected to CMS, followed by treatment with ASD or IMI. Data are mean ± standard error of the mean (SEM) (*n* = 8–11). ns, not significant, *p < 0.05, **p < 0.01, ***p < 0.001 versus Ctrl group, ^#^ *p* < 0.05, ^##^ *p* < 0.01, ^###^ *p* < 0.001 versus CMS group (one‐way ANOVA with Tukey's multiple‐comparisons test).

We first evaluated the anti‐depressant efficacy of ASD in mice (Figure 1A,B). The anhedonia of mice was evaluated by sucrose preference test (Figure 1C). We found that CMS mice displayed lower sucrose preference than control animals. Both ASD (40 or 80 mg/kg) and IMI (10 mg/kg) administration markedly increased sucrose preference in the CMS‐exposed mice (Figure 1C). The behavioral despair of mice was evaluated by forced swimming test (Figure 1D). The CMS mice displayed a shorter latency and longer immobility time in the forced swimming test. Both ASD (40 or 80 mg/kg) and IMI (10 mg/kg) treatments increased the latency and decreased the total duration of immobility in forced swimming test in the CMS‐exposed mice (Figure 1D). CMS decreased the time spent in center in open field test, which was reversed by 3‐week treatment with ASD (40 mg/kg; Figure 1E). In contrast, neither ASD nor IMI affected the distance traveled or immobility time in the open field test (Figure 1E). These results suggest that ASD ameliorates depressive‐like behaviors in CMS mice.

### 3.2 Akebia saponin D rescues CMS‐induced deficits in hippocampal neurogenesis

**FIGURE 2:**
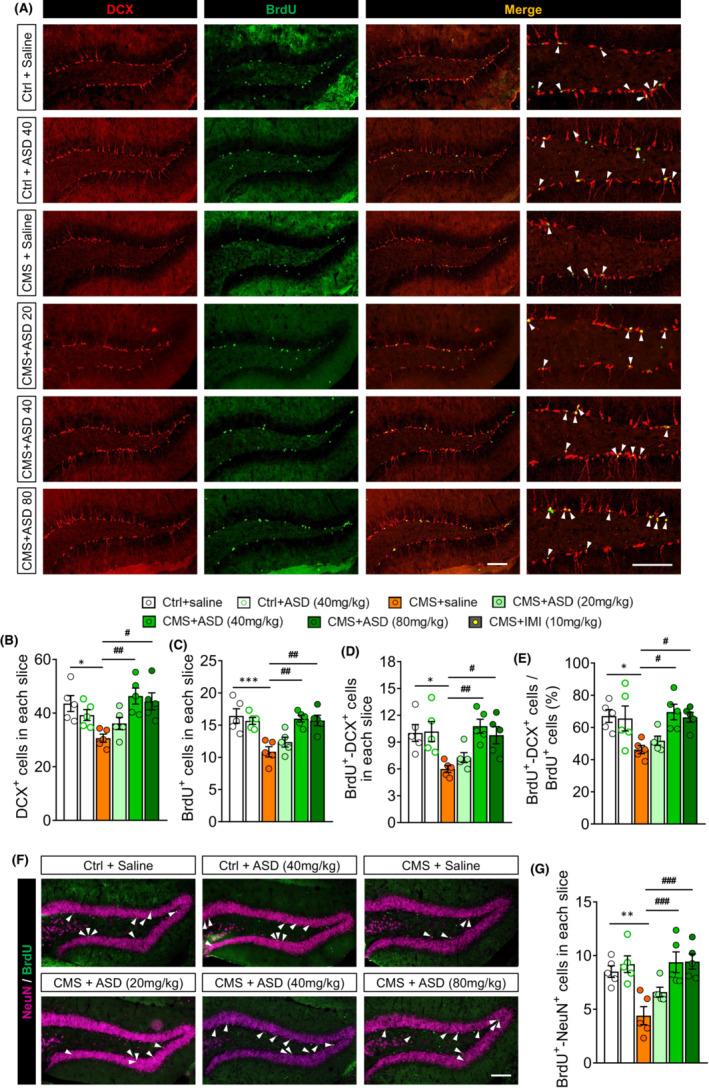
Akebia saponin D promotes hippocampal neurogenesis of CMS‐exposed mice. (A) Immunofluorescence micrographs of hippocampal newborn neurons (BrdU^+^–DCX^+^ cells), marked with arrowheads. Proliferating neural stem/precursor cells were labeled with 5′‐bromo‐2′deoxyuridine (BrdU, green), and immature neurons were labeled with doublecortin (DCX, red). Scale bar, 100 μm. (B) Quantification of hippocampal immature neurons (DCX^+^ cells). (C) Quantification of hippocampal proliferating neural stem/precursor cells (BrdU^+^ cells). (D) Quantification of hippocampal newborn neurons (BrdU^+^–DCX^+^ cells). (E) The percentage of NSPC differentiation into neuron (BrdU^+^–DCX^+^ cells out of BrdU^+^ cells). (F) Immunofluorescence micrographs of mature new neurons (BrdU^+^–NeuN^+^ cells) in hippocampus. Proliferating neural stem/precursor cells were labeled with 5′‐bromo‐2′deoxyuridine (BrdU, green), and mature neurons were labeled with neuron‐specific nucleoprotein (NeuN, pink). The BrdU^+^–NeuN^+^ cells were marked with arrowheads. Scale bar, 100 μm. (G) Quantification of mature new neurons (BrdU^+^–NeuN^+^ cells) in hippocampal dentate gyrus. Results for each group were obtained from five mice, for each of which five hippocampal slices were examined at 40× magnification. Each dot in the bar graph represents the average of all micrographs for one mouse. Data are mean ± standard error of the mean (SEM) (*n* = 5). *p < 0.05, **p < 0.01, ***p < 0.001 versus control (Ctrl) group, ^#^ *p* < 0.05, ^##^ *p* < 0.01, ^###^ *p* < 0.001 versus CMS group (one‐way ANOVA with Tukey's multiple‐comparisons test).

NSPCs in the dentate gyrus (DG) of the hippocampus proliferate and differentiate into neurons even in adulthood, and this neurogenesis is negatively associated with depression and positively associated with the efficacy of anti‐depressants.^9^, ^21^ Consistent with this, CMS strikingly reduced the number of newborn neurons (DCX^+^) in the hippocampus of our mice (Figure 2A,B). In fact, CMS reduced the numbers of BrdU^+^ cells and DCX^+^–BrdU^+^ cells and the rate of neuronal differentiation in the subgranular zone (SGZ) of the hippocampus, suggesting the inhibition of NSPC proliferation and neuronal differentiation (Figure 2C–E). However, ASD (40 or 80 mg/kg) reversed the CMS‐induced decrease in DCX^+^, BrdU^+^, and DCX^+^–BrdU^+^ cells, as well as the rate of neuronal differentiation (Figure 2C–E).

To examine the effects of ASD on the survival and maturation of proliferative cells, BrdU was injected into mice and the mature new neurons (BrdU^+^–NeuN^+^ cells) were examined (Figure 2F). CMS reduced the numbers of BrdU^+^–NeuN^+^ cells in the DG of hippocampus. ASD (40 or 80 mg/kg) treatment also reversed these effects of CMS on survival and maturation of proliferative cells (Figure 2G). These results indicate that ASD improves NSPC proliferation, survival, and neuronal differentiation and maturation in the hippocampus of CMS mice.

### 3.3 The anti‐depressant effects of akebia saponin D depend in part on promoting hippocampal neurogenesis

**FIGURE 3:**
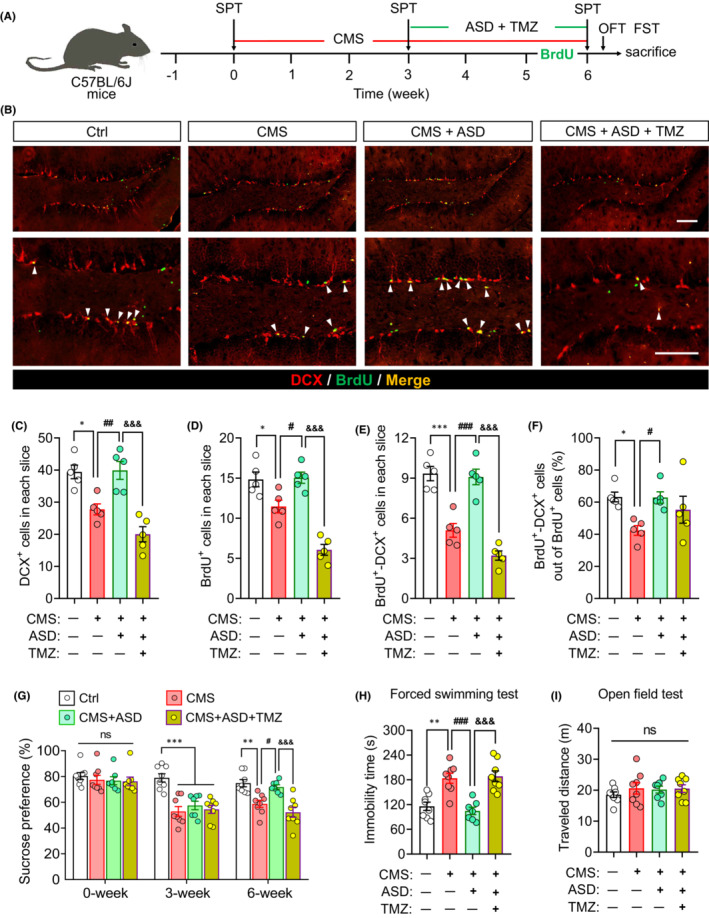
The anti‐depressant effects of akebia saponin D depend in part on promoting hippocampal neurogenesis. (A) Scheme of the experimental procedure for ablating neurogenesis with temozolomide (TMZ) in ASD‐treated CMS mice. ASD, akebia saponin D; CMS, chronic mild stress; FST, forced swimming test; OFT, open field test; SPT, sucrose preference test; TMZ, temozolomide. (B) Immunofluorescence micrographs of hippocampal newborn neurons (BrdU^+^–DCX^+^ cells), marked with arrowheads. Proliferating neural stem/precursor cells were labeled with 5′‐bromo‐2′deoxyuridine (BrdU, green), and immature neurons were labeled with doublecortin (DCX, red). Scale bar, 100 μm. (C) Quantification of hippocampal immature neurons (DCX^+^ cells). (D) Quantification of hippocampal proliferating neural stem/precursor cells (BrdU^+^ cells). (E) Quantification of hippocampal newborn neurons (BrdU^+^–DCX^+^ cells). (F) The percentage of NSPC differentiation into neuron (BrdU^+^–DCX^+^ cells out of BrdU^+^ cells). (G) Sucrose preference of mice on baseline (0 week), before treatment (3 weeks), or after treatment (6 weeks) with ASD in the absence or presence of TMZ. ns, not significant. (H) Changes in immobility time of CMS mice treated with ASD in the absence or presence of TMZ in the forced swimming test. (I) Changes in distance traveled by CMS mice treated with ASD in the absence or presence of TMZ in the open field test. ns, not significant. Results for each group were obtained from five mice for panel (C–F) and seven to eight mice for panel (G–I). Data are mean ± standard error of the mean (SEM). *p < 0.05, **p < 0.01, ***p < 0.001 versus the control (Ctrl) group; ^#^ *p* < 0.05, ^##^ *p* < 0.01, ^###^ *p* < 0.001 versus the CMS group; ^&^ *p* < 0.05, ^&&^ *p* < 0.01, ^&&&^ *p* < 0.001 versus the CMS + ASD group based on one‐way ANOVA with Tukey's multiple‐comparisons test.

To investigate the role of neurogenesis in antidepressant effects of ASD, we used the temozolomide (TMZ) to ablate neurogenesis in ASD‐treated CMS mice (Figure 3A). TMZ treatment significantly reduced the number of DCX^+^, BrdU^+^, and DCX^+^–BrdU^+^ cells, as well as the rate of neuronal differentiation in SGZ of hippocampus of CMS + ASD mice (Figure 3B–F). Ablation of neurogenesis using TMZ abolished the anti‐depressant effects of ASD in the sucrose preference test (Figure 3G) and forced swimming test (Figure 3H), but did not affect the traveled distances in open filed test (Figure 3I). These results suggest that the anti‐depressant effects of ASD depend in part on promoting hippocampal neurogenesis.

### 3.4 Akebia saponin D reprogrammes a pro‐neurogenic microglia in dentate gyrus of CMS mice

**FIGURE 4:**
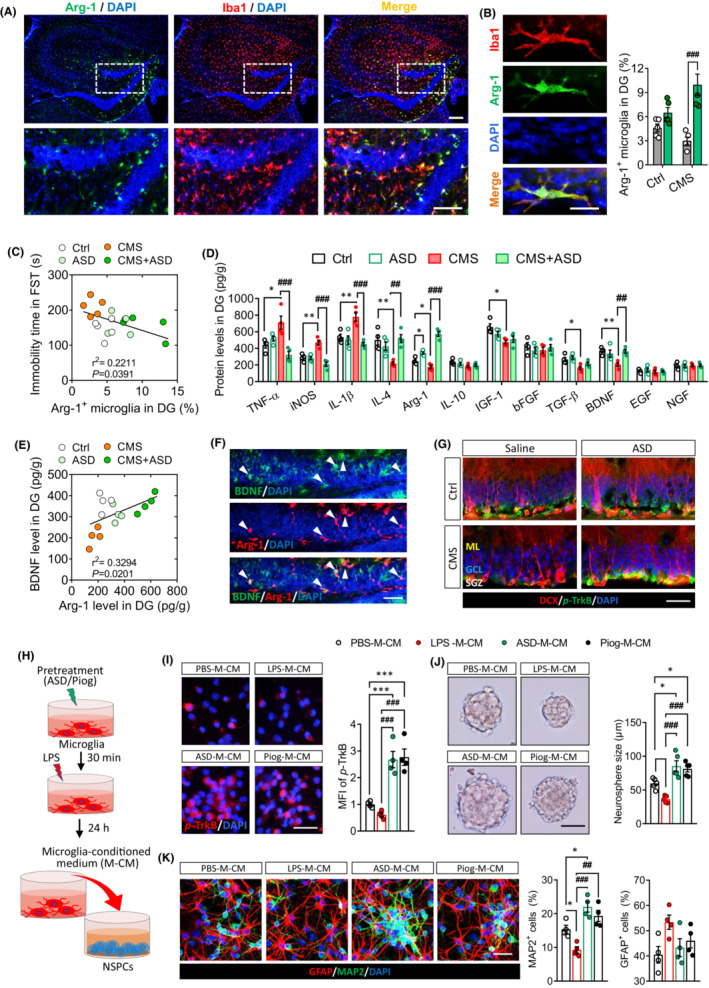
Akebia saponin D reprogrammes a pro‐neurogenic microglia phenotype in the dentate gyrus of CMS mice. (A) Immunofluorescence micrographs of Arg‐1^+^ microglia in the dentate gyrus of CMS mice after treatment with ASD. Scale bar, 100 μm. (B) Immunofluorescence micrographs and quantification of Arg‐1^+^ microglia in the dentate gyrus of control (Ctrl) or CMS mice after treatment with saline or ASD. Scale bar, 5 μm. (C) Correlation of immobility time in the forced swimming test with the percentage of Arg‐1^+^ microglia in dentate gyrus of Ctrl, ASD, CMS, and CMS + ASD mice. Each circle represents one mouse (*n* = 5). (D) Levels of pro‐ and anti‐inflammatory cytokines and neurotrophic factors in the dentate gyrus of Ctrl and CMS mice following ASD treatment. (E) Correlation of BDNF with Arg‐1 levels in dentate gyrus of Ctrl, ASD, CMS, and CMS + ASD mice. Each circle represents one mouse (*n* = 4). (F) Immunofluorescence micrographs of BDNF in Arg‐1^+^ microglia of the dentate gyrus of CMS + ASD mice. Scale bar, 50 μm. (G) Micrographs of *p*‐TrkB in the subgranular zone (SGZ) of hippocampus. Scale bar, 50 μm. (H) Scheme of the experimental procedure detailing the collection of conditioned medium from microglia activated with LPS in the presence or absence of ASD. The medium was then added to neural stem/progenitor cells (NSPCs) cultures for their proliferation, differentiation, and survival detection. (I) Micrographs and quantification of pTrkB in NSPCs incubated with conditioned medium (CM) from microglia that had been cultured normally (PBS‐M‐CM), in the presence of LPS (LPS‐M‐CM), or in the presence of ASD or Piog followed by LPS (ASD‐M‐CM, Piog‐M‐CM). Scale bar, 20 μm. (J) Micrographs and quantification of neurosphere size after incubation in conditioned medium from microglia. Scale bar, 100 μm. (K) Micrographs and quantification of pleiotropic NSPC differentiation in conditioned medium from microglia. Astrocytes were labeled with antibody against GFAP (green) and neurons with antibody against MAP2 (red). Scale bar, 50 μm. Panels (A–E): Results for each group were obtained from four to five mice, for each of which five hippocampal slices were examined at 40× magnification. Each dot in the bar graph represents the average of all micrographs for one mouse. Data are mean ± standard error of the mean (SEM). *p < 0.05, **p < 0.01 versus the Ctrl group; ^##^ *p* < 0.01, ^###^ *p* < 0.001 versus the CMS group based on two‐way ANOVA with Tukey's multiple‐comparisons test. Panels (I–K): Results of each group were obtained from four to six slides, and five micrographs (40×) were collected from each slide. The average of all micrographs in each slide was used for statistical analysis. Data are mean ± standard error of the mean (SEM). *p < 0.05, **p < 0.01, ***p < 0.001 versus PBS‐M‐CM group; ^#^ *p* < 0.05, ^##^ *p* < 0.01, ^###^ *p* < 0.001 versus LPS‐M‐CM group (one‐way ANOVA with Tukey's multiple‐comparisons test).

Microglia control the neurogenic microenvironment, and the Arg‐1^+^ microglia contribute to hippocampal neurogenesis.^9^ Therefore, we examined the effects of ASD on Arg‐1^+^ microglia in dentate gyrus of CMS‐exposed mice. The results showed that ASD significantly increased the percentage of Arg‐1^+^ microglia in dentate gyrus of CMS‐exposed mice (Figure 4A,B). The immobility time in forced swimming test was negatively correlated with Arg‐1^+^ microglia in dentate gyrus of mice (Figure 4C). ASD also reversed the CMS‐induced increases in the pro‐inflammatory factors TNF‐α, iNOS, and IL‐1β and decreases in the anti‐inflammatory factors IL‐4 and Arg‐1 in dentate gyrus of mice (Figure 4D). Analogously, CMS substantially reduced the levels of IGF‐1, TGF‐β, and BDNF, while ASD significantly increased BDNF in dentate gyrus of mice (Figure 4D). The BDNF levels were positively correlated with Arg‐1 levels in dentate gyrus of mice (Figure 4E). The results from immunofluorescent staining showed that ASD upregulated the BDNF in Arg‐1^+^ microglia in dentate gyrus (Figure 4F). Considering that ASD increases the microglial secretion of BDNF, which in turn promotes neurogenesis from NSPCs, we examined the levels of phosphorylation of the BDNF‐specific receptor TrkB in hippocampus of mice. The results showed that CMS reduced the levels of p‐TrkB in the SGZ of hippocampus, which ASD reversed (Figure 4G).

To confirm that ASD directly regulates microglial function, we examined the effects of ASD on primary cultures of microglia that were treated with LPS as a model of neuroinflammation (Figure S1). LPS shifts microglia toward a pro‐inflammatory phenotype that inhibits NSPC proliferation, survival, and differentiation.^21^ Pretreatment with ASD at 50 or 100 μM, but not 10 μM, prevented LPS from upregulating iNOS and TNF‐α and increased the expression of IL‐10, Arg‐1, and BDNF at 24 and 48 h (Figures S1 and S2).

To confirm that the ASD‐induced changes in microglia in turn influence NSPCs, we treated primary cultures of microglia in different ways, then transferred the culture medium to NSPC cultures, and observed their proliferation, survival, and neuronal differentiation (Figure 4H and Figure S3). We first examined the levels of p‐TrkB in primary NSPCs cultured in conditioned medium from microglia. The conditioned medium from microglia treated with 50 μM ASD + LPS (ASD‐M‐CM) increases the pTrkB in NSPCs (Figure 4I). Compared with conditioned medium from PBS‐treated microglia (PBS‐M‐CM), the conditioned medium from LPS‐treated microglia (LPS‐M‐CM) decreased the size of NSPC neurospheres. ASD‐M‐CM increased the size of NSPC neurospheres when compared with PBS‐M‐CM or LPS‐M‐CM (Figure 4J). LPS‐M‐CM inhibited the NSPC differentiation into neurons (DCX^+^ cells), but ASD‐M‐CM promoted such differentiation (Figure 4K). Overall, the effects of ASD were similar to those of pioglitazone, an agonist of PPAR‐γ pathway, which reprogrammes a pro‐neurogenic microglial phenotype.

### 3.5 The PPAR‐γ plays a critical role in reprogramming of pro‐neurogenic microglia by akebia saponin D in dentate gyrus of CMS mice

**FIGURE 5:**
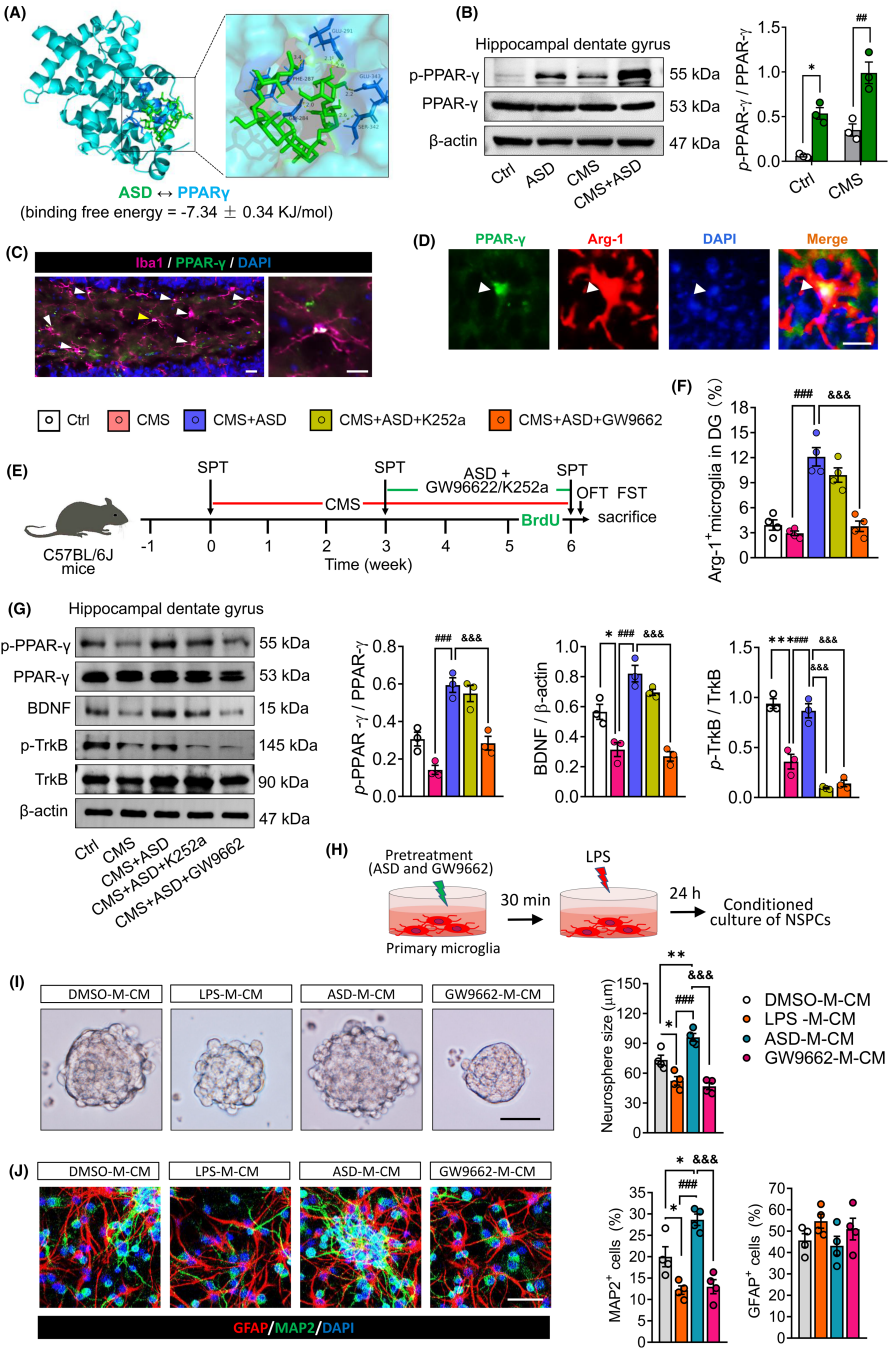
Blocking the PPAR‐γ signaling pathway abolished the pro‐neurogenic microglia induced by akebia saponin D in dentate gyrus of CMS mice. (A) Molecular docking of akebia saponin D (green) to peroxisome proliferator‐activated receptor (PPAR)‐γ. Key residues predicted to be involved in the complexes are shown in green for akebia saponin D and in blue for the binding partners. (B) Western blotting of PPAR‐γ and phosphorylated PPAR‐γ (*p*‐PPAR‐γ) in the dentate gyrus (DG) of hippocampus of control (Ctrl) or CMS mice after treatment with saline or ASD. Levels of *p*‐PPAR‐γ were normalized to those of PPAR‐γ (*n* = 3, each sample in triplicate). (C) Fluorescence micrographs showing PPAR‐γ expression in Iba1^+^ microglia of dentate gyrus in CMS mice after treatment with ASD. PPAR‐γ was stained with antibody (green), microglia were stained with an anti‐Iba1 antibody (pink), and nuclei were stained with DAPI (blue). The nuclear translocation of PPAR‐γ was indicated by arrowhead. Scale bar, 10 μm. (D) Fluorescence micrographs showing PPAR‐γ expression in Arg‐1^+^ microglia of dentate gyrus in CMS mice after treatment with ASD. PPAR‐γ was stained with antibody (green), Arg‐1^+^ microglia were stained with an anti‐Arg‐1 antibody (red), and nuclei were stained with DAPI (blue). The nuclear translocation of PPAR‐γ was indicated by white arrowhead. Scale bar, 10 μm. (E) Scheme of the experimental procedure detailing the blocking of PPAR‐γ or BDNF–TrkB signaling pathway in ASD + CMS mice. ASD, akebia saponin D; CMS, chronic mild stress; GW9662, PPAR‐γ inhibitor; FST, forced swimming test; K252a, TrkB inhibitor; OFT, open field test; SPT, sucrose preference test. (F) Effects of GW9662 or K252a treatment on the levels of Arg‐1^+^ microglia in dentate gyrus of ASD + CMS mice. (G) Effects of GW9662 or K252a treatment on levels of *p*‐PPAR‐γ, PPAR‐γ, BDNF, TrkB, and *p*‐TrkB in dentate gyrus of ASD + CMS mice (*n* = 3, each sample in triplicate). BDNF levels were normalized to those of β‐actin, while levels of *p*‐PPAR‐γ and *p*‐TrkB were normalized to those of PPAR‐γ and TrkB, respectively. (H) Scheme of the experimental procedure detailing the blocking of PPAR‐γ signaling pathway in ASD‐treated primary microglia. ASD, akebia saponin D; LPS, lipopolysaccharide. (I) Micrographs and quantification of neurosphere size after incubation in conditioned medium from dimethylsulfoxide‐treated microglia (DMSO‐M‐CM), lipopolysaccharide‐treated microglia (LPS‐M‐CM), LPS + ASD‐treated microglia (ASD‐M‐CM), and LPS + ASD + GW9662‐treated microglia (GW9662‐M‐CM). Scale bar, 100 μm. (J) Micrographs and quantification of NSPC differentiation incubated in conditioned medium from each group microglia. Scale bar, 30 μm. Data are mean ± standard error of the mean (SEM) (*n* = 3–6). Panel (A): *p < 0.05 versus Ctrl group; ^##^ *p* < 0.01 versus CMS group (two‐way ANOVA with Tukey's multiple‐comparisons test). Panels (F) and (G): *p < 0.05, ***p < 0.001 versus Ctrl group; ^###^ *p* < 0.001 versus CMS group; ^&&&^ *p* < 0.001 versus CMS + ASD group (one‐way ANOVA with Tukey's multiple‐comparisons test). Panels (I) and (J): *p < 0.05, **p < 0.01 versus DMSO‐M‐CM group; ^###^ *p* < 0.001 versus LPS‐M‐CM group; ^&&&^ *p* < 0.001 versus ASD‐M‐CM group (one‐way ANOVA with Tukey's multiple‐comparisons test).

Since mammalian target of PPAR‐γ signaling plays a key role in induction of anti‐inflammatory microglial phenotypes,^40^ we asked whether ASD acts via such signaling to exert its “microglial reprogramming” effect. Therefore, we explored the potential binding between ASD and PPAR‐γ (Figure 5A). Docking studies predicted the ligand ASD bound to the PPAR‐γ with a stability of −7.34 ± 0.34 kJ/mol (Figure 5A). Indeed, after treatment with ASD, p‐PPAR‐γ was significantly increased in hippocampal dentate gyrus of CMS‐exposed mice (Figure 5B). The results from immunofluorescent staining showed that PPAR‐γ localized in cytoplasm and nucleus of Arg‐1^+^ microglia in the dentate gyrus of mice that were exposed to CMS and then treated with ASD (Figure 5C,D).

To confirm the role of PPAR‐γ in induction of the pro‐neurogenic microglia in dentate gyrus of ASD‐treated mice, we repeated the above experiments in the presence of the PPAR‐γ antagonist GW9662 (Figure 5E), which effectively blocked the PPAR‐γ pathway in dentate gyrus (Figure 5G). Such blockade abolished the ability of ASD to increase the numbers of Arg‐1^+^ microglia in the dentate gyrus of CMS mice (Figure 5F). Blockade of PPAR‐γ signaling in ASD‐treated primary microglia also abolished the ability of ASD‐M‐CM to stimulate NSPC proliferation and neuronal differentiation (Figure 5H–J). These results suggest that PPAR‐γ plays a critical role in reprogramming of pro‐neurogenic microglia by akebia saponin D.

Activation of PPAR‐γ with ASD reversed the CMS‐induced decrease in BDNF and p‐TrkB levels in dentate gyrus of mice, which also abolished by GW9662 treatment (Figure 5G). Interestingly, the TrkB inhibitor K252a treatment effectively blocked the p‐TrkB, but did not affect the p‐PPAR‐γ and BDNF levels in dentate gyrus of CMS + ASD mice (Figure 5G). These results suggest that soluble microglial factors such as BDNF activate the TrkB of NSPC to promote NSPC proliferation, survival, and neurogenesis. Consistent with this, the TrkB inhibitor K252a prevented ASD‐M‐CM from stimulating NSPC proliferation and neuronal differentiation (Figure S4).

**FIGURE 6:**
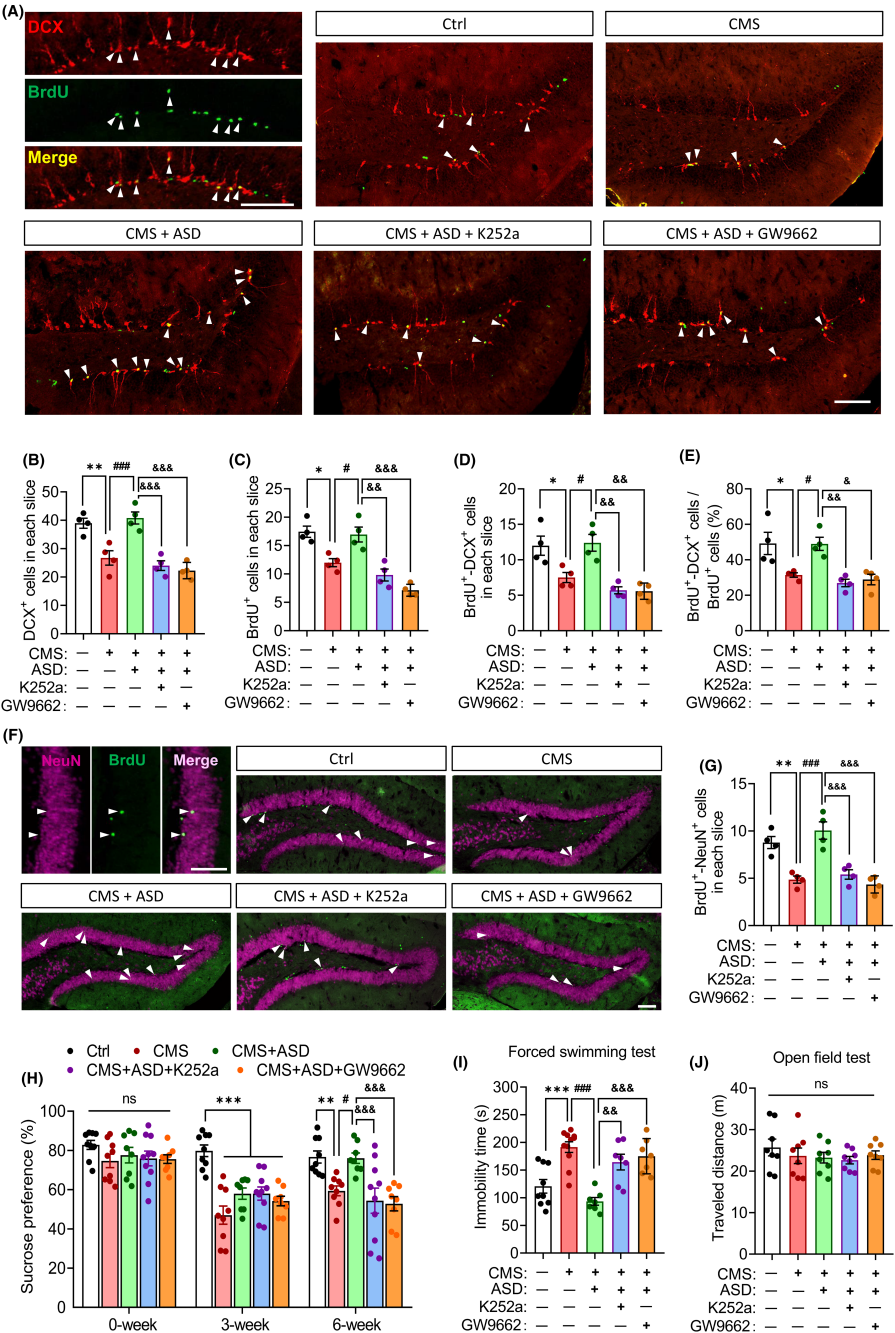
The pro‐neurogenic and anti‐depressant effects of akebia saponin D depend on the PPAR‐γ pathway. (A–E) Effects of K252a or GW9662 on the pro‐neurogenic effects of akebia saponin D in CMS‐exposed mice. Proliferating neural stem/precursor cells were labeled with 5′‐bromo‐2′deoxyuridine (BrdU, green), and immature neurons were labeled with doublecortin (DCX, red). Scale bar, 100 μm. The BrdU^+^‐DCX^+^ cells were indicated by white arrowheads. The hippocampal immature neurons (DCX^+^ cells), proliferating neural stem/precursor cells (BrdU^+^ cells), newborn neurons (BrdU^+^‐DCX^+^ cells), and percentage of BrdU^+^–DCX^+^ cells out of BrdU^+^ cells were quantized. Results for each group were obtained from four mice, for each of which five hippocampal slices were examined at 40× magnification. Each dot in the bar graph represents the average of all micrographs for one mouse. (F and G) Effects of K252a or GW9662 on the maturation of hippocampal newborn neurons in ASD + CMS mice. Proliferating neural stem/precursor cells were labeled with 5′‐bromo‐2′deoxyuridine (BrdU, green), and mature neurons were labeled with neuron‐specific nucleoprotein (NeuN, pink). The BrdU^+^–NeuN^+^ cells were marked with arrowheads. Scale bar, 100 μm. (H–J) Effects of K252a or GW9662 on the anti‐depressant effects of akebia saponin D (*n* = 8–10). Anhedonia, behavioral despair, and spontaneous activity were assessed separately in sucrose preference test, forced swimming test, and open field test. Data are mean ± standard error of the mean (SEM). *p < 0.05, **p < 0.01, ***p < 0.001 versus the control (Ctrl) group; ^#^ *p* < 0.05, ^##^ *p* < 0.01, ^###^ *p* < 0.001 versus the CMS group; ^&^ *p* < 0.05, ^&&^ *p* < 0.01, ^&&&^ *p* < 0.001 versus the CMS + ASD group based on one‐way ANOVA with Tukey's multiple‐comparisons test.

Blockade of PPAR‐γ or TrkB signaling abolished the ability of ASD to promote hippocampal neurogenesis in CMS mice (Figure 6A–G). Either GW9662 or K252a also blocked the anti‐depressant effects of ASD in the sucrose preference test and forced swimming test (Figure 6H–J). These results suggest that the anti‐depressant and pro‐neurogenic effects of akebia saponin D depend in part on PPAR‐γ and BDNF–TrkB signaling pathway.

## 4 DISCUSSION

Our previous research revealed that modulation of microglial phenotype and function may be an effective neurotherapy for depression.^9^, ^41^ Consistent with reports that natural products can be effective modulators of microglial phenotype and promoters of neurogenesis,^21^ here we demonstrate in vivo and in vitro that ASD, the major active ingredient in the traditional Chinese medicine *Dipsacus asper* Wall., can induce a pro‐neurogenic microglial phenotype in a PPAR‐γ‐dependent manner, which activates the BDNF–TrkB pathway in NSPCs to promote their proliferation and neuronal differentiation. The resulting neurogenesis can ameliorate depressive‐like behaviors in CMS mice.

We previously reported that ASD at 40 mg/kg significantly ameliorated depressive‐like behaviors in LPS‐treated mice.^32^ Here we found similar effects of ASD in mice exposed to CMS, a classical model of depression.^42^ In fact, ASD exerted similar anti‐depressant effects as imipramine, a commonly used clinical antidepressant.^43^ Decreased neurogenesis in the hippocampus is involved in the pathogenesis of depression and Alzheimer's disease^6^, ^44^, ^45^, ^46^ and has been associated with depressive‐like behaviors.^11^, ^47^ CMS decreased hippocampal neurogenesis in our mice, which ASD reversed, leading to increases in the numbers of DCX^+^, BrdU^+^, BrdU^+^–DCX^+^ and BrdU^+^–NeuN^+^ cells in the DG, where NSPCs undergo proliferation and neuronal differentiation.^21^, ^48^, ^49^, ^50^ The resulting neurons are known to participate in mood and behavior.^51^, ^52^ These results indicate that ASD rescues CMS‐induced deficits in hippocampal neurogenesis by promoting NSPC proliferation, survival, and neuronal differentiation and maturation.

Psychological stress activates microglia to secrete pro‐inflammatory factors that impair neuroplasticity, especially in the dentate gyrus of hippocampus.^53^ Consistent with this, CMS strongly increased the number and area of microglia in the hippocampus of our mice, and it upregulated pro‐inflammatory TNF‐α and IL‐1β, while downregulating the anti‐inflammatory factors IL‐4 and Arg‐1 and neurotrophic factors IGF‐1, TGF‐β, and BDNF. ASD reversed all these changes, exerting a neuroprotective microglial phenotype in response to CMS. This result is consistent with our previous work,^32^ where we implicated the nuclear transcription factor PPAR‐γ in the effects of ASD on microglial phenotype. Consistent with that study, we found here that the PPAR‐γ agonist pioglitazone, like ASD, induced an anti‐inflammatory microglial phenotype. Our findings here may help explain how ASD can attenuate microglia‐mediated inflammation in animal models of depression.^32^, ^33^

The strong ability of NSPCs to proliferate and differentiate makes them promising targets for repairing nerve injury.^54^ However, adverse changes in the microenvironment of the CNS, including signals from microglia,^55^ can induce NSPCs to differentiate into astrocytes at the expense of neurons, which increases the risk of glial scar formation.^21^ A pro‐inflammatory phenotype of microglia, which can be induced in animal models using CMS or LPS,^56^ is thought to suppress adult NSPC proliferation.^21^ Here, we also found that factors secreted by microglia exposed to LPS inhibited differentiation of adult NSPCs into neurons. Pretreating microglia with ASD, in contrast, enabled microglia to promote NSPC proliferation, survival, and neuronal neurogenesis. Thus, ASD appears to induce secretion of neurogenic factors from microglia, which then influence NSPCs. This mechanism may explain how ASD can exert anti‐depressant effects and mitigate cognitive impairment in animal models of depression.^30^, ^32^

One of the neurogenic factors secreted by ASD‐treated microglia appears to be BDNF, which plays a neuroprotective and neurotrophic role.^57^, ^58^, ^59^ We found that ASD treatment upregulated BDNF in hippocampal microglia of CMS mice. Pretreating microglia with ASD before LPS increased the secretion of BDNF into the medium, such that this conditioned medium promoted NSPC proliferation and neuronal differentiation. ASD also activated the BDNF receptor, TrkB, in the SGZ of CMS mice. Consistent with a role of BDNF as mediator of ASD‐induced neurogenesis, we found that NSPC proliferation and differentiation correlated with an increase in levels of p‐TrkB, while the TrkB inhibitor K252a blocked ASD‐induced neurogenesis and anti‐depressant effects.

We found that the ability of ASD to reprogramme pro‐neurogenic microglial phenotype is mediated by PPAR‐γ, a ligand‐dependent transcription factor belonging to the nuclear hormone receptor superfamily.^60^ PPAR‐γ regulates the expression of anti‐inflammatory cytokines,^61^ and the PPAR‐γ agonists pioglitazone or rosiglitazone can switch activated microglia cells from a pro‐inflammatory to anti‐inflammatory state.^62^ Our previous research showed that ASD acts via PPAR‐γ to switch activated microglia from a pro‐inflammatory to anti‐inflammatory phenotype in vitro.^33^ In present study, we further demonstrated that ASD acts via PPAR‐γ to induce a pro‐neurogenic microglial phenotype in dentate gyrus of CMS‐exposed mice and mitigate depressive‐like mouse behaviors. Conversely, blocking the PPAR‐γ signaling pathway abolished the Arg‐1^+^ microglia and BDNF expression induced by ASD in dentate gyrus of CMS‐exposed mice, as well as the pro‐neurogenic and antidepressant effects of ASD. Drugs may have potential effects on NGF receptor TrkA,^63^ and subsequent exploration of BND‐TrkB targeting inhibitors is needed.

**FIGURE 7:**
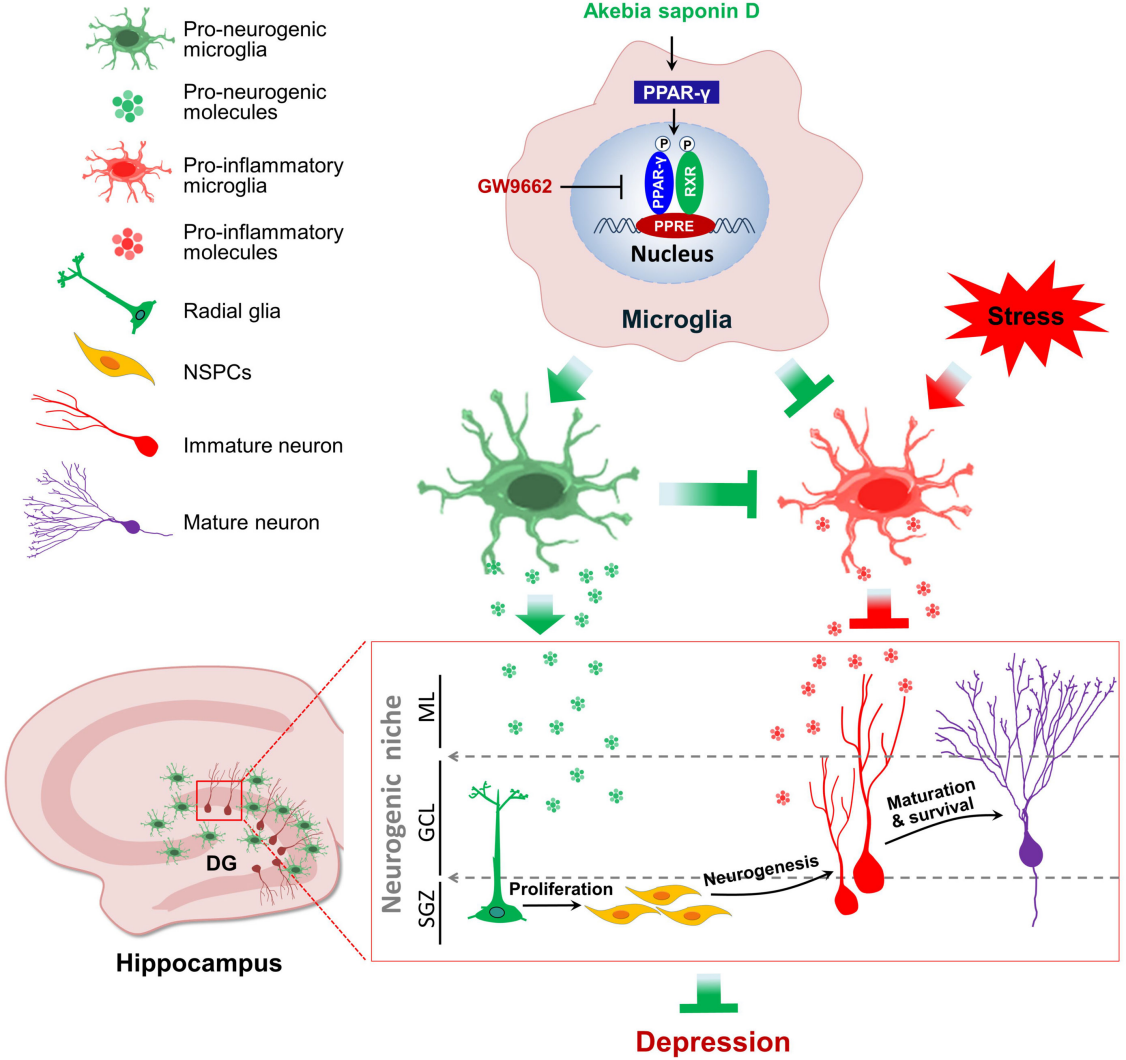
Schematic diagram of how akebia saponin D may restore hippocampal neurogenesis. Akebia saponin D acts via the PPAR‐γ pathway to reprogramme a pro‐neurogenic microglial phenotype in dentate gyrus that can increase BDNF expression which to promote NSPC proliferation, survival, and neural differentiation, thus restores hippocampal neurogenesis and ameliorates depression‐like behavior in CMS‐exposed mice.

Taken together, these experiments strongly suggest that ASD acts via the PPAR‐γ pathway to reprogramme a pro‐neurogenic microglia in dentate gyrus of CMS mice that can increase BDNF expression and promote NSPC proliferation, survival, and neuronal differentiation (Figure 7). This neurogenesis then mitigates CMS‐induced deficits in hippocampal neurogenesis and depressive‐like behaviors. Our results justify further studies of ASD as a potential treatment for depression and may inspire new lines of research targeting the PPAR‐γ pathway in disorders involving impaired neurogenesis.

## AUTHOR CONTRIBUTIONS

JZ, TZ, and ZY conceived and designed the study. JZ and QL wrote the manuscript, which was revised by TZ and ZY, approved by all the authors. QL, CX, and HH performed behavioral tests and immunostaining. LL cultured NSPCs and performed immunofluorescence and cytokine assays. DS performed statistical analyses of the data.

## FUNDING INFORMATION

This work was supported by the National Natural Science Foundation of China (82060726), the Department of Science and Technology of Guizhou High‐level Innovative Talents ([2018]5638‐2) and Sichuan Science and Technology Program (2020YJ0225).

## CONFLICT OF INTEREST STATEMENT

The authors declared no potential conflicts of interest with respect to the research, authorship, and/or publication of this article.

## Supporting information


Figures S1–S4.

Tables S1–S3.

## Data Availability

The data that support the findings of this study are available from the corresponding author upon reasonable request.
