# Supplementary material for: Akebia saponin D acts via the PPAR‐gamma pathway to reprogramme a pro‐neurogenic microglia that can restore hippocampal neurogenesis in mice exposed to chronic mild stress
Source: CNS Neurosci Ther. 2023 Mar 29;29(9):2555–71. doi: 10.1111/cns.14196 (PMC10401137; doi:10.1111/cns.14196)
Supplement: Supplementary file 1 — Figures S1–S4. [file CNS-29-2555-s001.docx]

**Supplementary figures**

| 1 | Supplementary Figure 1 | Akebia saponin D regulates the microglial phenotype in LPS stimulation |
| --- | --- | --- |
| 2 | Supplementary Figure 2 | Akebia saponin D stimulate LPS-treated microglia to release BDNF |
| 3 | Supplementary Figure 3 | Experimental scheme for the assessment of NSPCs proliferation, differentiation and survival during culture in conditioned medium from microglia |
| 4 | Supplementary Figure 4 | Involvement of the BDNF-TrkB pathway in the ASD-induced pro-neurogenic phenotype of microglia |


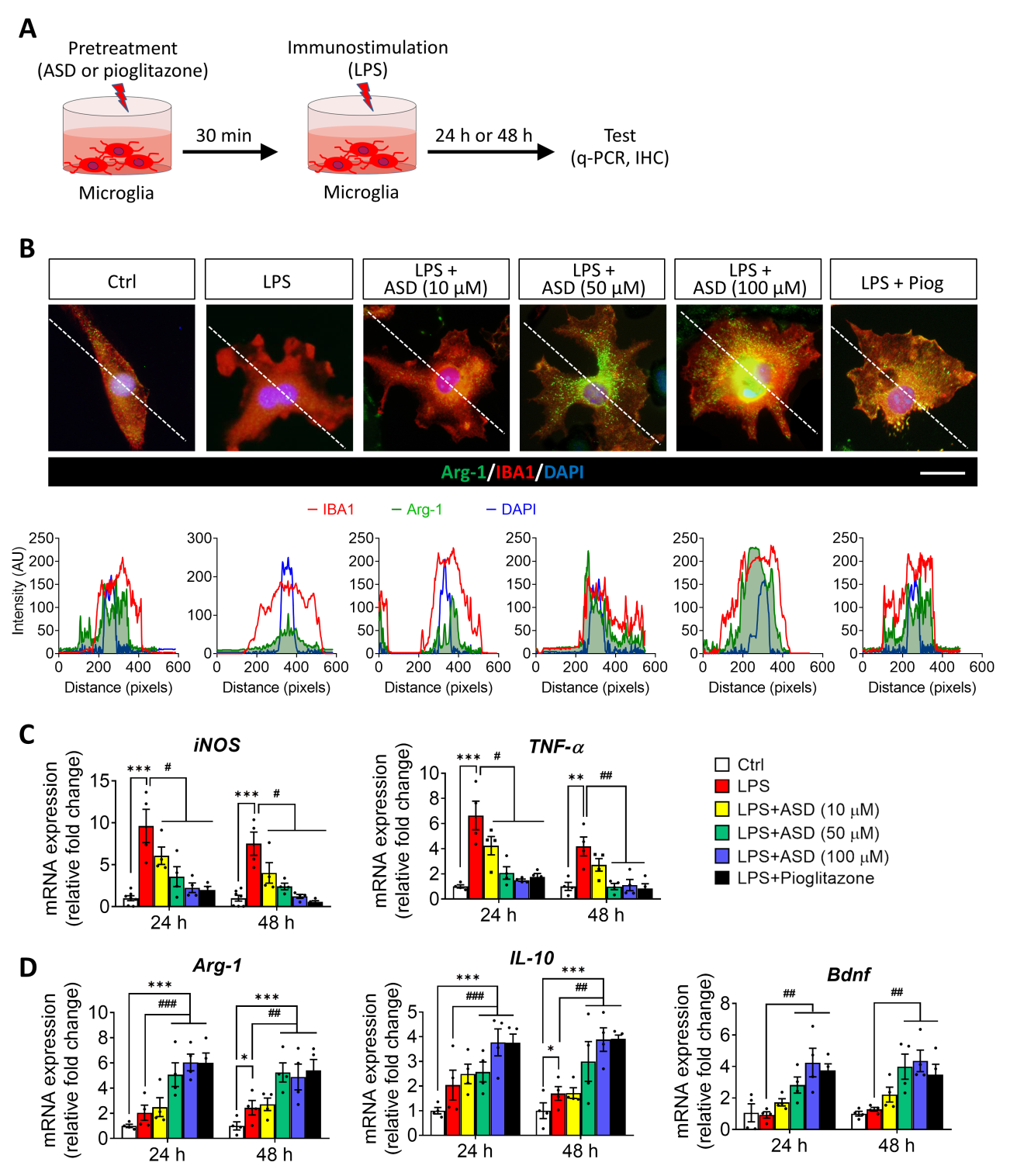


**Fig. S1 Akebia saponin D regulates the microglial phenotype in LPS stimulation.**

**(A)** Scheme of the experimental procedure for examining microglial anti-inflammatory phenotype. ASD, akebia saponin D; LPS, lipopolysaccharide; qPCR, real-time quantitative PCR; IHC, immunocytochemistry.

**(B)** Morphology and Arg-1 expression in primary microglia treated with akebia saponin D (ASD) or pioglitazone (Piog), followed by lipopolysaccharide (LPS). Scale bar, 10 μm. The corresponding peaks at the bottom of the micrograph represent the dotted-lines indicating the changes in fluorescence density of IBA1, Arg-1 and DAPI.

**(C)** and **(D)** Levels of mRNAs encoding iNOS, TNF-α, Arg-1, IL-10 and BDNF at 24 and 48 h in microglia treated with ASD or pioglitazone followed by LPS.

Data are mean ± standard error of the mean (SEM) (n=4-6). *p < 0.05, **p < 0.01, ***p < 0.001 vs. control (Ctrl); ^#^p < 0.05, ^##^p < 0.01, ^###^p < 0.001 vs. LPS based on two-way ANOVA with Tukey's multiple-comparisons test. The results of statistical analyses are listed in supplementary Table 10.


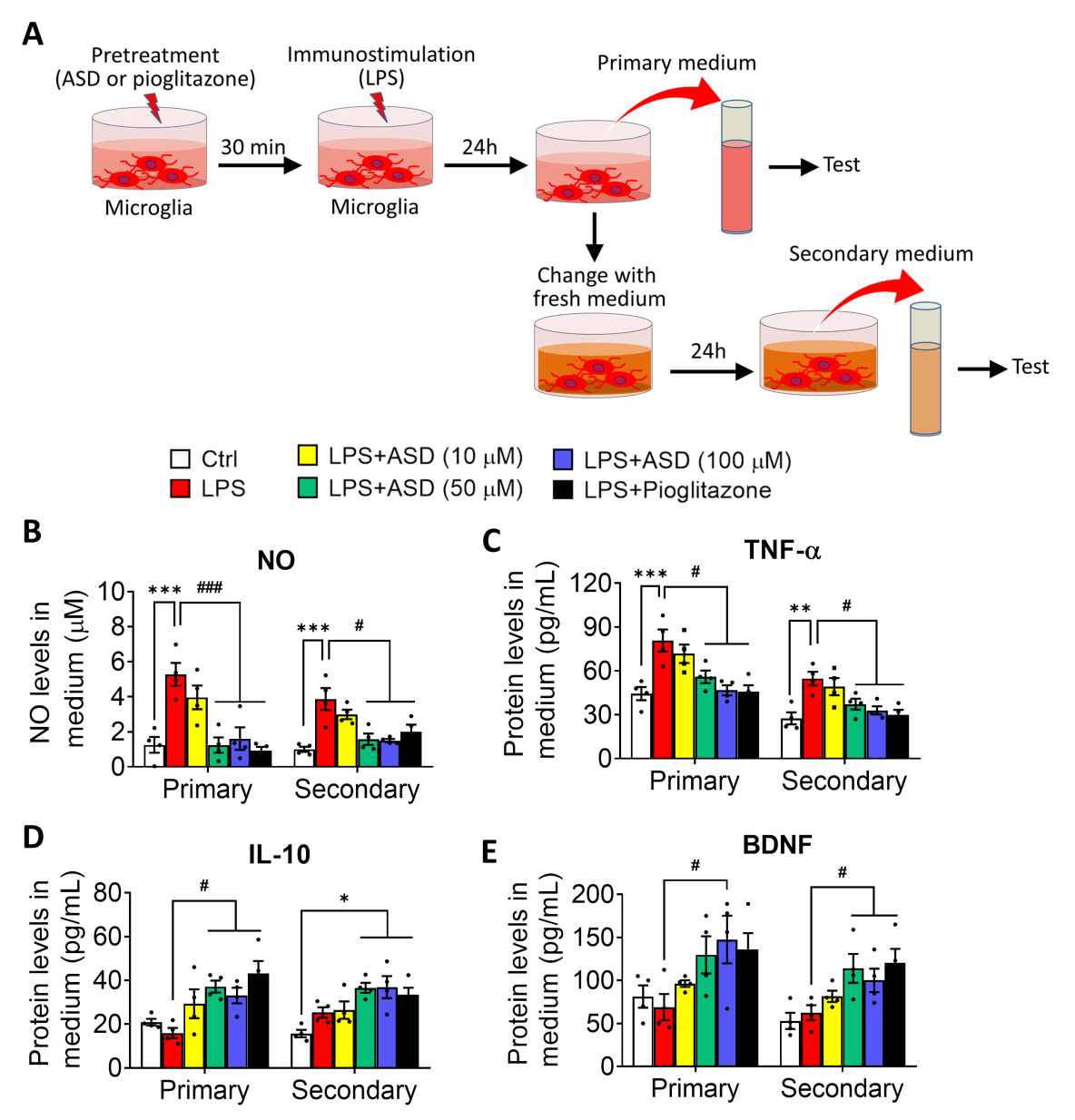


**Fig. S2 Akebia saponin D stimulate LPS-treated microglia to release BDNF.**

**(A)** Schematic diagram of the collection of conditioned medium at 0-24 h (“primary”) and 24-48 h (“secondary”) from primary cultures of microglia activated with LPS in the presence or absence of ASD.

**(B-E)** Quantification of TNF-α, nitric oxide (NO), IL-10 and BDNF in conditioned medium from microglia.

Data are mean ± standard error of the mean (SEM) (n=4-6). *p < 0.05, ***p < 0.001 vs. control (Ctrl); ^#^p < 0.05, ^###^p < 0.001 vs. LPS based on two-way ANOVA with Tukey's multiple-comparisons test. The results of statistical analyses are listed in supplementary Table 11.


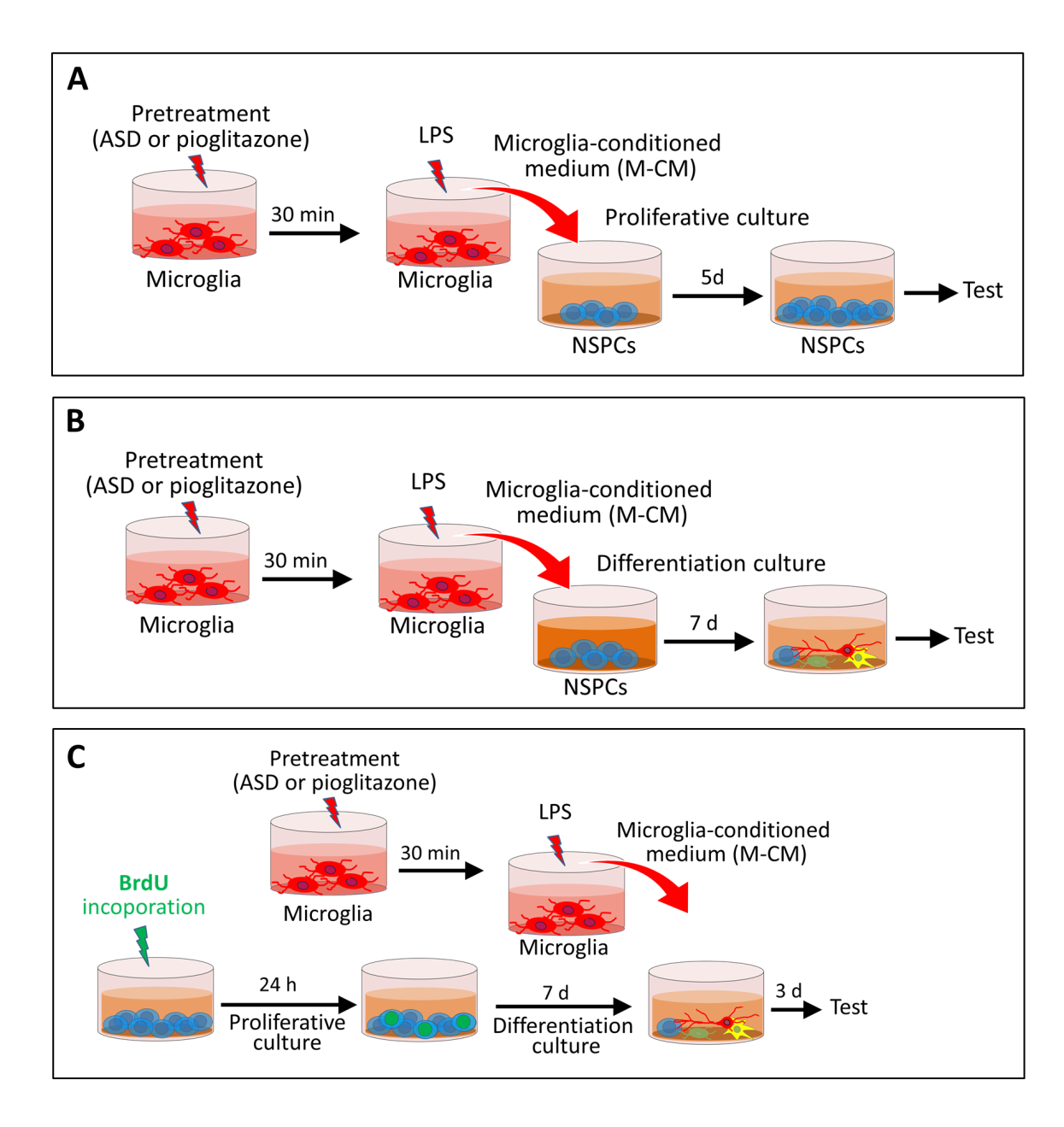


**Fig. S3 Experimental scheme for the assessment of NSPCs proliferation, differentiation and survival during culture in conditioned medium from microglia.**

**(A**) Experimental scheme for the assessment of NSPCs proliferation during culture in conditioned medium from microglia activated with LPS in the presence or absence of ASD.

**(B**) Experimental scheme for the assessment of NSPCs differentiation during culture in conditioned medium from microglia activated with LPS in the presence or absence of ASD.

**(C**) Experimental scheme for the assessment of NSPCs and newborn neurons survival during culture in conditioned medium from microglia activated with LPS in the presence or absence of ASD.


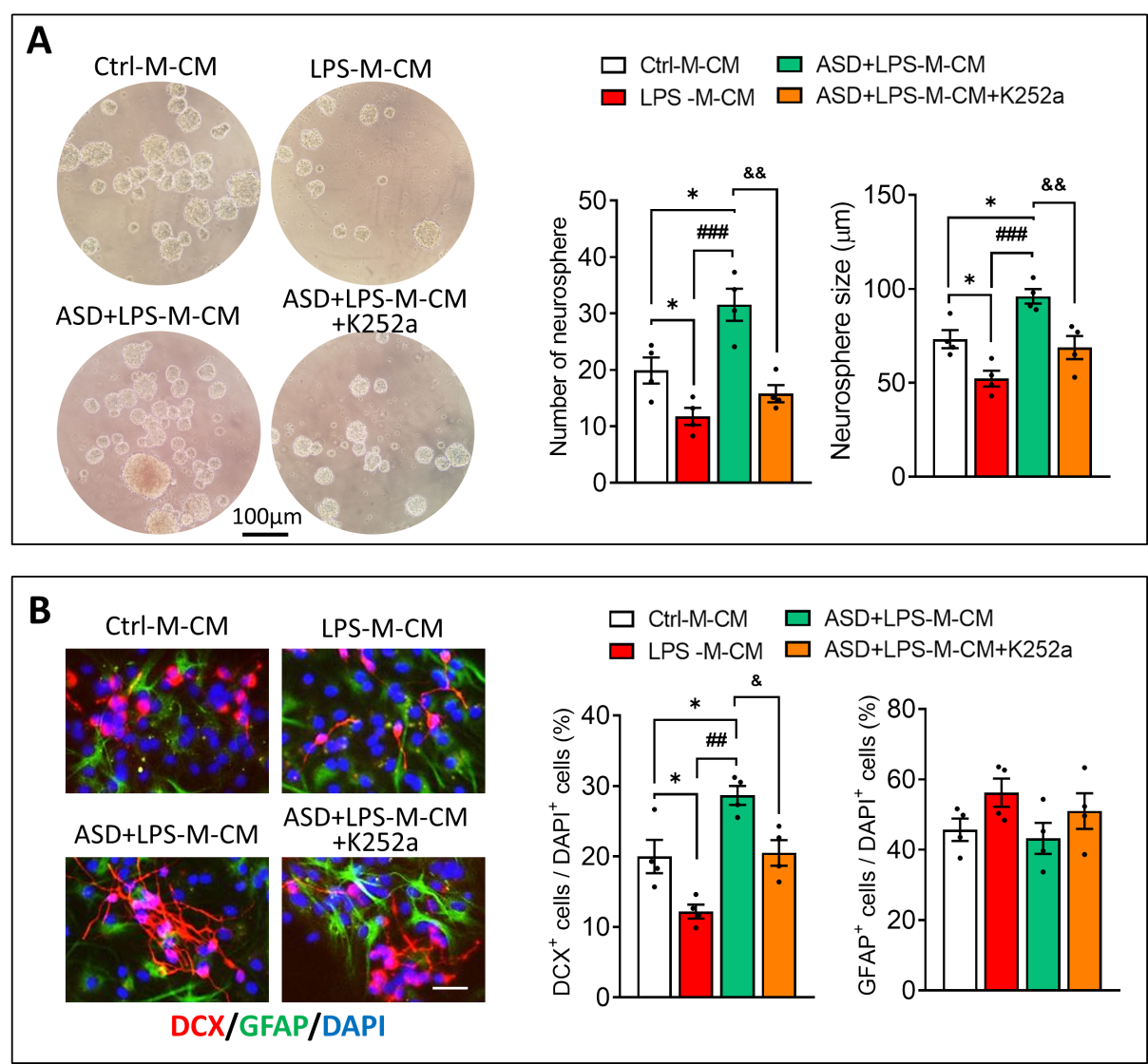


**Fig. S4 Involvement of the BDNF-TrkB pathway in the ASD-induced pro-neurogenic phenotype of microglia.**

**(A)** Effects of K252a on proliferation of NSPCs cultured in conditioned medium from microglia. Scale bar, 100 μm.

**(B**) Effects of K252a on neurogenesis and astrocytogenesis from NSPCs cultured in conditioned medium from ASD-induced microglia. Scale bar, 20 μm.

Data are presented as the mean ± standard error of the mean (SEM) (n=4-6), **P* < 0.05 vs. control microglia conditioned medium (Ctrl-M-CM), ^###^*P* < 0.001 vs. LPS-treated microglia conditioned medium (LPS-M-CM), ^&^*P* < 0.05, ^&&^*P* < 0.01 vs. ASD + LPS-treated microglia conditioned medium (ASD + LPS-M-CM) by one-way ANOVA with Tukey's multiple-comparisons test. The results of statistical analyses are listed in supplementary Table 12.
