# Supplementary material for: Akebia saponin D acts via the PPAR‐gamma pathway to reprogramme a pro‐neurogenic microglia that can restore hippocampal neurogenesis in mice exposed to chronic mild stress
Source: CNS Neurosci Ther. 2023 Mar 29;29(9):2555–71. doi: 10.1111/cns.14196 (PMC10401137; doi:10.1111/cns.14196)
Supplement: Supplementary file 2 — Tables S1–S3. [file CNS-29-2555-s002.docx]

**Supplementary Tables**

| 1 | Supplementary Table 1 | Genes primers used for real time PCR analyses |
| --- | --- | --- |
| 2 | Supplementary Table 2 | Details of Antibody and dilution rate for immunofluorescent staining |
| 3 | Supplementary Table 3 | Details of Antibody and dilution rate for western blotting |

**Supplementary Table 1. Genes primers used for real time PCR analyses**

| **Gene** | **Primer sequences** |
| --- | --- |
| *β-actin* | Forward: 5’-CCGTGAAAAGATGACCCAGATC-3’  Reverse: 5’-CACAGCCTGGATGGCTACGT-3’ |
| *Tnf-α* | Forward:5’-TACTGAACTTCGGGGTGATTGGTCC-3’  Reverse: 5’-CAGCCTTGTCCCTTGAAGAGAACC-3’ |
| *iNOS* | Forward: 5’-ACAACAGGAACCTACCAGCTCA-3’  Reverse: 5’-GATGTTGTAGCGCTGTGTGTCA-3’ |
| *IL-10* | Forward: 5’-TGGCCCAGAAATCAAGGAGC-3’  Reverse: 5’-CAGCAGACTCAATACACACT-3’ |
| *Arg-1* | Forward: 5’-AGACAGCAGAGGAGGTGAAGAG-3’  Reverse: 5’-CGAAGCAAGCCAAGGTTAAAGC-3’ |
| *Bdnf* | Forward: 5’-GAGCTGAGCGTGTGTGACAG-3’  Reverse: 5’-CGCCAGCCAATTCTCTTTTTGC-3’ |

**Supplementary Table 2. Details of Antibody and dilution rate for immunofluorescent staining**

| AntiBody | Manufacturer | Product numbers | Dilution Rate |
| --- | --- | --- | --- |
| Iba1 (Goat anti-mouse) | Abcam | ab178846 | 1:400 |
| GFAP (Mouse anti-mouse) | Cell Signaling Technology | 3670S | 1:400 |
| BrdU (Mouse anti-mouse) | Cell Signaling Technology | 5292S | 1:400 |
| DCX (Rabbit anti-mouse) | Cell Signaling Technology | 14802S | 1:300 |
| BDNF (Rabbit anti-mouse) | Abcam | ab108319 | 1:500 |
| p-TrkB (Rabbit anti-mouse) | Invitrogen | PA5-38076 | 1:50 |
| MAP2 (Rabbit anti-mouse) | Cell Signaling Technology | 4542S | 1:400 |
| Arg-1 (Mouse anti-mouse) | Abcam | ab239731 | 1:200 |
| PPAR-γ (Rabbit anti-mouse) | Cell Signaling Technology | 2435S | 1:300 |
| NeuN (Rabbit anti-mouse) | Cell Signaling Technology | 36662SF | 1:800 |

**Supplementary Table 3. Details of Antibody and dilution rate for western blotting**

| Antibody | Manufacturer | Product numbers | Dilution Rate |
| --- | --- | --- | --- |
| TrkB (Rabbit anti-mouse) | Cell Signaling Technology | 4603S | 1:1000 |
| p-TrkB (Rabbit anti-mouse) | Invitrogen | PA5-38076 | 1:500 |
| PPAR-γ (Rabbit anti-mouse) | Cell Signaling Technology | 2435S | 1:800 |
| p-PPARγ (S112) (Rabbit anti-mouse) | ImmunoWay Biotechnology | YP0316 | 1:500 |
| BDNF (Rabbit anti-mouse) | Abcam | ab108319 | 1:800 |
| β-actin (Mouse anti-mouse) | Abcam | ab6276 | 1:1000 |
